# High frequency of viridians group streptococci bacteremia in pediatric neuroblastoma high-risk patients during induction chemotherapy

**DOI:** 10.1038/s41598-023-31805-3

**Published:** 2023-04-06

**Authors:** Ola El Kebbi, Cassandra S. Prather, Lena Elmuti, Malak Khalifeh, Muayad Alali

**Affiliations:** 1 Department of Emergency Medicine, American University of Beirut Medical Center, Beirut, Lebanon; 2 Department of Pediatrics, Indiana University School of Medicine, Indianapolis, IN USA; 3 Department of Pediatrics, Pediatric Hematology-Oncology, University of Chicago Medicine, Chicago, IL USA; 4 Department of Pediatrics, Ryan White Center for Pediatric Infectious Diseases and Global Health, Indiana University School of Medicine, 705 Riley Hospital Drive, RI-5862, Indianapolis, IN 46202 USA

**Keywords:** Cancer, Microbiology, Medical research, Oncology

## Abstract

Existing literature on febrile neutropenia (FN) has categorized patients with acute leukemia or those undergoing allogeneic stem cell transplantation (SCT) as being high risk for severe infection, bacteremia, and poor outcomes. Comprehensive studies of infection risk in pediatric high-risk neuroblastoma (NB-HR) during induction chemotherapy are limited, and mostly merged within the solid tumor (ST) group. Therefore, it is unclear whether infectious complications and outcomes for NB-HR are the same as in other ST groups. We conducted a retrospective medical record review of pediatric FN patients in a single center from March 2009 to December 2016. FN episodes were categorized into five groups based on underlying diagnosis (acute myelogenous leukemia (AML), acute lymphocytic leukemia (ALL), NB-HR during induction chemotherapy, other solid tumors, and SCT). Comparative analyses of infectious complications between patients with NB-HR and those with other types of cancer diagnoses were performed. A total of 667 FN episodes (FNEs) were identified in 230 patients. FNEs occurred in 82 episodes with NB-HR. Bloodstream infection (BSI) occurred in 145 (21.7%) of total FN episodes. The most isolated organisms were the viridians group streptococci (VGS) (25%). NB-HR patients have higher rates of VGS bacteremia (OR 0.15, 95% [CI 0.04, 0.56]) and are more likely to be admitted to the Pediatric Intensive Care Unit (PICU) compared to patients with other solid tumors (OR 0.36, 95% [CI 0.15, 0.84]). Interestingly, there is no difference in VGS rates between patients with NB-HR and those with AML despite the fact that NB-HR patients do not receive a cytosine arabinoside (AraC)-based regimen. This large neuroblastoma cohort showed that patients with NB-HR during induction chemotherapy are at higher risk for VGS bacteremia and PICU admissions compared with patients with other solid tumors. Further prospective studies are needed to investigate infection-related complications in this high-risk group and to improve morbidity and mortality.

## Introduction

Neuroblastoma (NB) is the most prevalent childhood solid extracranial tumor in children and the third most common childhood cancer, accounting for around 15% of all pediatric cancer deaths^1^. Among infants younger than 12 months, NB is twice as common as leukemia^2^. In the United States alone, more than 600 cases are diagnosed every year and 50% are considered high-risk due to the adverse features of the tumor or an advanced disease course. The international neuroblastoma risk group (INRG) classifies high-risk NB (NB-HR) as tumors with MYCN amplification, metastatic tumors in children over 18 months, or in those less than 18 months with 11q aberrations^3^. The current chemotherapy regimen for NB-HR spans over 18 months and includes induction, consolidation [tandem autologous stem cell transplantation (SCT)], and maintenance therapy. The induction phase alone includes 5 to 8 cycles of intensive chemotherapy with platinum and alkylating agents^4^. Due to the high intensity of treatment, infectious complications are very common among this patient population.

It has been established that central line-associated bloodstream infections (CLABSIs) are associated with significant morbidity and mortality in pediatric cancers, particularly leukemia^5–8^. Over the past 10–15 years, VGS has accounted for 25 to 30% of bacteremia episodes among cancer patients and is the most common etiology for early BSI in recipients of allogeneic hematopoietic stem cell transplantation (HSCT)^9–11^. VGS is a commensal organism present in the gastrointestinal tract and female genital tract^8^. In the immunocompromised host, VGS could result in fever, hypotension, severe pulmonary compromise, as well as manifestations of viridans-related septic shock syndrome (VSSS)^12^. The risk of VSSS is more pronounced among pediatric patients with VGS bacteremia compared to their adult counterparts^13^ with fatality rates reaching as high as 12.5%^12,14–16^. Neutropenic oncology patients who have severe mucositis after receiving intensive chemotherapy are more at risk of developing VGS bacteremia^17^. Acute myelogenous leukemia (AML) patients have historically been known to be at higher risk for VGS bacteremia compared to patients with other malignancies, given that they receive high dose cytosine arabinoside (HD-AraC); however, studies looking at VGS bacteremia in NB-HR during induction chemotherapy are lacking limited.

Currently, most of the pediatric oncology literature focuses on infectious risk and bacteremia in acute leukemias and SCT recipients, with a paucity in the NB-HR population during induction chemotherapy. In most studies, NB-HR has been grouped with other solid tumors, for which the treatment regimens are often not as intensive. The question of whether the infectious risk in NB-HR patients during induction chemotherapy should be compared to patients with other solid tumors remains unclear^18^. Moreover, most of the available literature on NB-HR is from large clinical trials that lack information on the types of pathogens and infections encountered^19–22^.

In our study, we evaluated the spectrum of pathogens from isolated blood cultures in patients with NB-HR during induction chemotherapy. Our primary endpoint was to compare the VGS bacteremia rates in NB- HR patients during induction chemotherapy to that of bacteremia rates in patients with other solid tumors, acute lymphocytic leukemia (ALL), AML, and SCT recipients, and to define risk factors associated with VGS bacteremia in this group. Our secondary endpoint was to compare FN-associated morbidity and mortality in patients NB-HR during induction chemotherapy to that of patients with other diagnoses.

## Methods

### Study design and population

A retrospective medical record review was conducted on pediatric patients 21 years of age or younger from March 2009 to December 2016 who received oncologic care at the University of Chicago Medicine (UCM) Comer Children’s Hospital. The need for informed consent was formally waived by the Institutional Review Board of the University of Chicago. All methods were carried out in accordance with relevant guidelines and regulations. The experimental protocol was approved by the Institutional Review Boards at the University of Chicago. The need for informed consent was formally waived by the approving committee. The Clinical Research Data Warehouse was queried for hospitalized patients with FN from March 2009 to December 2016 with an International Classification for Disease-9th edition (ICD-9) or ICD-10 code. A retrospective electronic medical record (EMR) review was performed to verify that FN episodes were appropriate for inclusion based on the below characteristics. All episodes not meeting the above-mentioned criteria were excluded. Data for all variables listed below were extracted by EMR review. For patients with more than one admission for FN, each admission was counted as a separate episode. Patients included were pediatric oncology patients with different underlying diagnoses (ALL, AML, NB-HR during induction chemotherapy, other solid tumors (ST), or SCT recipients) who were hospitalized for FN (absolute neutrophil count (ANC) < 500/mm^3^) and had at least one blood culture obtained.

For analysis purposes, the following diseases were grouped together based on similar chemotherapy intensity: mixed leukemia and AML, ALL regardless of disease status, Hodgkin’s and non-Hodgkin’s lymphoma, and autologous and allogeneic SCT recipients. The term “NB-HR” in this paper referred only to high-risk neuroblastoma high risk during induction chemotherapy. FN episodes in NB-HR patients who underwent autologous transplants (during consolidation and maintenance) were grouped with other SCT recipients. Low and intermediate-risk neuroblastoma patients were excluded from this study. Also, febrile non-neutropenic episodes post-transplant were excluded. Patients with incomplete charts and those in remission or not actively receiving chemotherapy were excluded as well. There were no major changes during the study period in either empirical therapy and management in FN or NB-HR chemotherapy during induction. We separated NB-HR from all other solid tumors which included a heterogeneous group of tumors such as Wilms tumor, osteosarcoma, hepatoblastoma, Ewing sarcoma, rhabdomyosarcoma, intracranial malignancies, etc.

### Definitions

Fever was defined as any recorded temperature ≥ 38.0 °C (≥ 100.4°F). Neutropenia was defined as an ANC of less than 500/mm^3^, thrombocytopenia was defined as a platelet of less than 50 per microliter^23^. Positive blood culture results (bacteria, fungal) were classified as a true pathogen (i.e., bloodstream infection) or a contaminant using the National Health Safety Network (NHSN) criteria for skin commensals^23^. IFD was stratified as possible, probable, and proven according to the latest European Organization for Research and Treatment of Cancer-Invasive Fungal Infections Cooperative Group/National Institute of Allergy and Infectious Diseases Mycosis Study Group (EORTC/MSG) criteria (2020). Mucositis was categorized into 5 grades based on National Cancer Institute/Common Toxicity Criteria (CTC) criteria; we included only only mucositis grade ≥ 2 was included in this study. FN-related PICU admissions were defined as the need to transfer to the PICU within 7 days of FN presentation^24^. Neuroblastoma high-risk patients were defined based International Neuroblastoma Risk Group (INRG) classification system. Patients who were already located in the PICU prior to FN onset were not counted as PICU admission. FN-associated mortality was defined as within 30 days from FN onset.

### Clinical patient management

Pediatric FN patients were managed per standard institutional practice, which did not undergo any major changes during the study period. Ceftazidime is the initial empiric antimicrobial for FN patients with vancomycin ± gentamicin added based on clinical presentation (i.e., concern for central venous catheter infection or septic shock). Cefepime was administered instead of ceftazidime for selected patients with high-risk FN such as AML. Antibacterial prophylaxis was not routinely used except Trimethoprim-sulfamethoxazole for Pneumocystis jirovecii pneumonia (PJP) prophylaxis. Empiric antifungal therapy was added if the patient remained febrile on day 5 of antibiotics and if neutropenia was expected to last longer than 5 to 7 days.

### Statistical analysis

SPSS version 27 was used for data analysis. Data were presented as frequency and percentages for categorical variables or as means and standard deviations for continuous variables. Chi-squared test or Fisher’s exact test was used when appropriate to compare different underlying cancer diagnoses. A p-value less than 0.05 was considered statistically significant.

Logistic regression was used to study the association between different outcomes and underlying cancer diagnoses and for adjusting to adjust for confounding variables including age, gender, ethnicity, ANC, absolute monocyte count (AMC), absolute lymphocyte count (ALC), platelet count, as well as chemotherapy within the last 2 weeks or during the FN episode. In this analysis, NB-HR was considered as the reference group in comparison to other types of underlying cancer.

## Results

**Figure 1:**
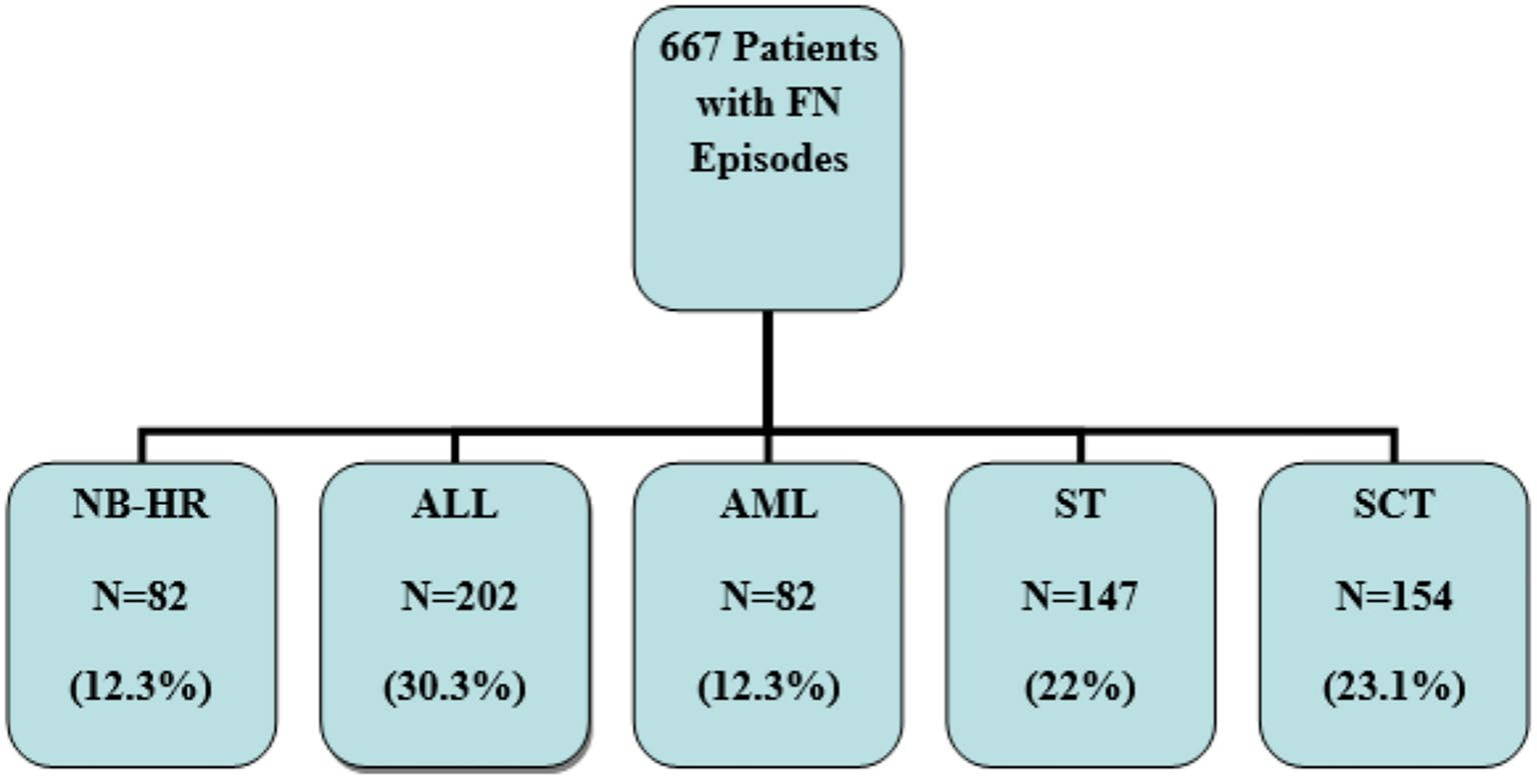
Distribution of types of malignancies among patients with FN.

A total of 667 FN episodes were included in the study. About half were males (54.7%) and older than 10 years old (55.8%). They had different underlying cancer diagnoses including NB-HR (12.3%), ALL (30.3%), AML (12.3%), other ST (22%), and SCT (23.1%) (Fig. 1). Patients with NB-HR were primarily under 10 years old (80.5%) and males (54.7%) (Table 1).

**Table 1:** Baseline and clinical characteristics of cancer patients.

Option	Total n = 667 (%)	NB N = 82 (12.3%)	ALL N = 202 (30.3%)	AML N = 82 (12.3%)	Solid tumor N = 147 (22%)	SCT N = 154 (23.1%)	p-value
Age							
< 10 years	295 (44.2%)	66 (80.5%)	88 (43.6%)	37 (45.1%)	50 (34.0%)	54 (35.1%)	< 0.001
> 10 years	372 (55.8%)	16 (19.5%)	114 (56.4%)	45 (54.9%)	97 (66.0%)	100 (64.9%)	
Gender							
Male	365 (54.7%)	50 (61.0%)	92 (45.5%)	51 (62.2%)	81 (55.1%)	91 (59.1%)	0.024
Female	302 (45.3%)	32 (39.0%)	110 (54.5%)	31 (37.8%)	66 (44.9%)	63 (40.9%)	
Ethnicity							
Unknown	29 (4.3%)	0 (0.0%)	0 (0.0%)	12 (14.6%)	6 (4.1%)	11 (7.1%)	< 0.001
Non-Hispanic	492 (73.8%)	64 (78.0%)	151 (74.8%)	52 (63.4%)	117 (79.6%)	108 (70.1%)	
Hispanic	146 (21.9%)	18 (22.0%)	51 (25.2%)	18 (22.0%)	24 (16.3%)	35 (22.7%)	
Chemotherapy within the last 2 weeks	481 (72.1%)	71 (86.6%)	148 (73.3%)	52 (63.4%)	117 (79.6%)	93 (60.4%)	< 0.001
Fever > 39 °C	172 (25.8%)	27 (32.9%)	48 (23.8%)	24 (29.3%)	29 (19.7%)	44 (28.6%)	0.16
GI associated symptoms	230 (34.5%)	42 (51.2%)	72 (35.6%)	21 (25.6%)	62 (42.2%)	33 (21.4%)	< 0.001
Mucositis	178 (26.7%)	39 (47.6%)	37 (18.3%)	47 (32.0%)	15 (18.3%)	40 (26%)	< 0.001
URI	205 (30.7%)	27 (32.9%)	78 (38.6%)	16 (19.5%)	53 (36.1%)	31 (20.1%)	< 0.001
Chills	38 (5.7%)	5 (6.1%)	12 (5.9%)	7 (8.5%)	5 (3.4%)	9 (5.8%)	0.605
Platelet < 50	418 (62.7%)	57 (69.5%)	105 (52%)	64 (78.0%)	87 (59.2%)	105 (68.2%)	< 0.001
History BSI	200 (30%)	16 (19.5%)	53 (26.2%)	40 (48.8%)	28 (19%)	63 (40.9%)	< 0.001
Low BP	103 (15.4%)	29 (35.4%)	21 (10.4%)	17 (20.7%)	14 (9.5%)	22 (14.3%)	< 0.001
RVP positive							
Negative	378 (56.7%)	42 (51.2%)	115 (56.9%)	49 (59.8%)	84 (57.1%)	88 (57.1%)	0.055
Positive	127 (19.0%)	12 (14.6%)	49 (24.3%)	8 (9.8%)	29 (19.7%)	29 (18.8%)	
Unknown	162 (24.3%)	28 (34.1%)	38 (18.8%)	25 (30.5%)	34 (23.1%)	37 (24.0%)	
ANC							
< 100	466 (69.9%)	64 (78.0%)	123 (60.9%)	64 (78.0%)	117 (79.6%)	98 (63.6%)	< 0.001
> 100	201 (30.1%)	18 (22.0%)	79 (39.1%)	18 (22.0%)	30 (20.4%)	56 (36.4%)	
AMC							
< 100	497 (76.1%)	69 (85.2%)	148 (75.5%)	68 (86.1%)	110 (75.9%)	102 (67.1%)	0.005
> 100	156 (23.9%)	12 (14.8%)	48 (24.5%)	11 (13.9%)	35 (24.1%)	50 (32.9%)	
ALC							
< 100	252 (38.3%)	41 (50.6%)	45 (22.6%)	31 (38.3%)	45 (31.0%)	90 (59.2%)	< 0.001
> 100	406 (61.7%)	40 (49.4%)	154 (77.4%)	50 (61.7%)	100 (69.0%)	62 (40.8%)	

*NB* neuroblastoma, *ALL* acute lymphoblastic leukemia, *AML* acute myelogenous leukemia, *SCT* stem cell transplant, *GI* gastrointestinal, *URI* upper respiratory tract infection, *BSI* bloodstream infection, *BP* blood pressure, *RVP* respiratory virus panel, *ANC* absolute neutrophil count, *AMC* absolute monocyte count, *ALC* absolute lymphocyte count.

Most of the included patients in this study received chemotherapy within the last 2 weeks or during the FN episode (72.1%). Gastrointestinal symptoms were the most common associated symptoms (34.5%), followed by upper respiratory tract infection (URI) symptoms (30.7%), mucositis (26.7%), and chills (5.7%). Additionally, 62.7% had low platelet levels and 21% had true bacteremia. Most of them had an ANC < 100/mm^3^ (69.9%) and an AMC < 100/mm^3^ (76.1%). Most patients with NB-HR had received chemotherapy within the last 2 weeks or during the FN episode (86.6%). Among patients with NB-HR, 51.2% had gastrointestinal symptoms, 32.9% had fever (> 39 °C) at the time of presentation, 32.9% had URI symptoms, 47.6% had mucositis, and 6.1% had chills. Table 2 presents the different outcomes of FN patients with NB-HR in comparison to those with other cancer diagnoses.

**Table 2:** Outcomes of febrile neutropenia episodes in patients with neuroblastoma compared to those with other underlying cancer diagnoses.

	Total n = 667 (%)	NB N = 82 (%)	ALL N = 202 (%)	AML N = 82 (%)	Solid tumor N = 147 (%)	SCT N = 154 (%)	p-value
BSI	145 (21.7%)	22 (26.8%)	39 (19.3%)	29 (35.4%)	23 (15.6%)	32 (20.8%)	
Crude OR (95%CI)		1	0.65 (0.36–1.19)	1.49 (0.77–2.91)	0.51 (0.26–0.98)*	0.72 (0.38–1.34)	0.008
Adjusted OR (95%CI)		1	0.71 (0.38–1.34)	1.54 (0.77–3.1)	0.57 (0.29–1.11)	0.71 (0.37–1.37)	0.021
VGS	37 (5.5%)	10 (12.2%)	9 (4.5%)	8 (9.8%)	3 (2%)	7 (4.5%)	
Crude OR (95%CI)		1	0.34 (0.13–0.86)*	0.78 (0.29–2.08)	0.15 (0.04–0.56)*	0.34 (0.13–0.94)*	0.016
Adjusted OR (95%CI)		1	0.43 (0.16–1.13)	1 (0.35–2.83)	0.17 (0.04–0.64)*	0.41 (0.14–1.18)	0.031
GP	74 (11.1%)	14 (17.1%)	21 (10.4%)	14 (17.1%)	5 (3.4%)	20 (13%)	
Crude OR (95%CI)		1	0.56 (0.27–1.17)	1 (0.44–2.26)	0.17 (0.06–0.49)*	0.73 (0.35–1.52)	0.01
Adjusted OR (95%CI)		1	0.65 (0.30–1.38)	1.09 (0.47–2.53)	0.19 (0.07–0.55)*	0.75 (0.35–1.64)	0.018
GN	56 (8.4%)	7 (8.5%)	14 (6.9%)	13 (15.9%)	10 (6.8%)	12 (7.8%)	
Crude OR (95%CI)		1	0.8 (0.31–2.06)	2.02 (0.76–2.35)	0.78 (0.29–2.14)	0.91 (0.34–2.4)	0.153
Adjusted OR (95%CI)		1	0.99 (0.37–2.65)	2.45 (0.88–2.796)	0.99 (0.35–2.79)	1.08 (0.39–3)	0.19
PICU	88 (13.2%)	14 (17.1%)	21 (10.4%)	14 (17.1%)	10 (6.8%)	29 (18.8%)	
Crude OR (95%CI)		1	0.56 (0.27–1.17)	1 (0.44–2.26)	0.36 (0.15–0.84)*	1.13 (0.56–2.28)	0.015
Adjusted OR (95%CI)		1	0.56 (0.26–1.20)	0.98 (0.41–2.31)	0.38 (0.16–0.93)*	1.14 (0.54–0.93)	0.032
Mortality	15 (2.2%)	1 (1.2%)	1 (0.5%)	3 (3.7%)	2 (1.4%)	8 (5.2%)	
Crude OR (95%CI)		1	0.40 (0.03–6.52)	3.08 (0.31–30.2)	1.12 (0.1–12.5)	4.44 (0.55–36.12)	0.091
Adjusted OR (95%CI)		1	0.28 (0.02–4.82)	1.81 (0.17–19.39)	1.06 (0.09–12.65)	2.41 (0.27–21.39)	0.33

*p-value < 0.05.

Odds ratio was adjusted for chemotherapy in the last 2 weeks or during the febrile neutropenia episode, fever, gastrointestinal symptoms, and mucositis.

*NB* neuroblastoma, *ALL* acute lymphoblastic leukemia, *AML* acute myelogenous leukemia, *SCT* stem cell transplant recipients, *BSI* bloodstream infection, *GP* gram positive, *GN* gram negative, *PICU* pediatric intensive care unit.

### Patient outcomes

#### Bacteremia

One hundred forty-five positive blood cultures were identified as pathogens in the 667 FN episodes (21%). AML was the most common underlying cancer associated with bloodstream infections (BSI) compared with other types of cancer. Patients with NB-HR during induction chemotherapy had significantly higher rates of BSI compared with other solid tumors (OR 0.51, 95% CI 0.26–0.98). Additionally, BSI rates for both ALL and SCT groups were noted to be lower than in the NB group (OR 0.65, 95% CI [0.36, 1.19] and OR 0.72, 95% CI [0.38, 1.34], respectively).

#### VGS bacteremia

VGS was the most commonly isolated pathogen (37/145, 25% of all positive blood cultures) as shown in Table 3. Ten out of 37 cases of VGS bacteremia occurred in NB-HR patients during induction chemotherapy, and four had VSSS. VGS bacteremia rates in the NB-HR group were 3 times higher than in the ALL group (OR 0.34, 95% CI [0.13, 0.86]) and in SCT recipients (OR 0.34, 95% CI [0.13, 0.94]), and 7 times higher than in other ST patients (OR 0.15, 95% CI [0.04–0.56]). NB-HR during induction chemotherapy had slightly higher VGS bacteremia rates compared with AML, but this was not statistically significant (OR 0.78, 95% CI [0.29, 2.08]). Patients with NB-HR during induction chemotherapy also had higher rates of gram-positive (GP) BSI when compared with those with other STs (OR 0.17, 95% CI [0.06, 0.49]). After further adjusting for variables including fever, chemotherapy, mucositis, and gastrointestinal symptoms, the NB-HR during the induction chemotherapy cohort continued to have statistically significant greater VGS bacteremia rates than the patients with other STs.

**Table 3:** Distribution of types of pathogens during BSI.

Bacterial microorganism	Frequency
VGS	37
Staphylococcus aureus	15
Staphylococcus epidermidis	30
Bacillus species	10
Enterococcus species	7
Gram negative bacilli	60
Other pathogens	12
Total pathogens in blood cultures	171
Total bacteremia events	145 (131 monomicrobial, 14 polymicrobial)

*BSI* bloodstream infection, *VGS* Viridans group streptococci.

#### PICU admission

PICU admission was required in 88/667 (13.2%) of FN episodes, and 14/88 (17%) were in NB-HR during induction chemotherapy. We found FNEs in NB-HR were more likely to be admitted to the PICU compared to other ST and ALL patients with FN (OR 0.36, 95% CI [0.15, 0.84] and OR 0.56, 95% CI [0.27, 1.17], respectively). There was no difference in PICU admissions compared with AML (OR 1.95% CI [0.44, 2.26]) and SCT recipients (OR 1.13, 95% CI [0.56, 2.28]). When the data was fit into a multivariate logistic regression model with adjusted variables, NB-HR during induction chemotherapy still had significantly higher rates of PICU admission compared with those with other solid tumors.

#### Mortality

Mortality associated with FN in this study was observed in only 15/667 (2.2%) FNEs, so there was no power in each category to conduct a meaningful analysis. There was no difference in mortality rates between NB-HR and other ST patients (OR 1.12, 95% CI [0.1, 12.5]), however, NB-HR during induction chemotherapy had a lower mortality rate than both AML patients (OR 3.08, 95% CI [0.31, 30.2]) and SCT recipients (OR 4.44, 95% CI [0.55, 36.12]), although these findings did not reach statistical significance.

#### Predictors of VGS infection

A multivariate logistic regression analysis was conducted to study predictors of VGS infection as shown in Table 4. The variables that fit the model included the type of malignancy and mucositis. It was found that patients with other solid tumors were less likely to have VGS bacteremia (aOR 0.167; 95% CI [0.044, 0.630]; p = 0.008) in comparison to patients with NB-HR. On the other hand, patients with mucositis are twice as likely to have VGS bacteremia [aOR 2.1; 95% CI [1.041, 4.297]; p = 0.038) as those without mucositis.

**Table 4:** Logistic regression: predictors of streptococcus infections.

	p-value	aOR	95% CI for EXP(B)	
Cancer type (compared to NB-HR)	0.029			
ALL	0.081	0.422	0.160	1.114
AML	0.977	0.985	0.356	2.727
Solid tumor	0.008	0.167	0.044	0.630
SCT	0.081	0.403	0.145	1.119
Mucositis	0.038	2.115	1.041	4.297

Odds ratio was adjusted for chemotherapy in the last 2 weeks or during the febrile neutropenia episode, fever, gastrointestinal symptoms, and mucositis. Omnibus test = 0.005, R square = 0.072, Hosmer and Lemeshow test: 0.578 (Backward LR logistic regression model). *ALL* acute lymphoblastic leukemia, *AML* acute myelogenous leukemia, *SCT* stem cell transplant.

## Discussion

In this large cohort study of pediatric FN, we examined the burden, risk factors, and outcomes of bacteremia in pediatric patients with NB-HR. We found that NB-HR patients have higher rates of VGS bacteremia and are more likely to be admitted to the PICU compared to patients with other solid tumors. This finding indicates the importance of stratifying infectious risks in NB-HR during induction chemotherapy compared to patients with other solid tumors in both future studies and clinical practice, which will help guide the approach to managing infections in this patient population.

In this study, 26.8% of patients with NB-HR during induction chemotherapy were found to have a BSI (all of them bacterial infection) during induction chemotherapy. Although this finding is lower than the 36% incidence of bacteremia that has been previously reported among pediatric NB-HR patients by Castagnola et al., this difference in rates may be explained by the fact that our study investigated bacteremia in FN episodes during induction therapy in this patient population, while Castagnola et al. included all NB-HR patients throughout their entire treatment course (including during consolidation with high-dose chemotherapy followed by autologous stem cell rescue)^25^. The few other studies that have looked at infectious complications in induction therapy for NB-HR have used varying definitions for infections^26,27^. Whittle et al. report nearly 40% (including 9% fungemia) of NB-HR during induction had at least one BSI, and nearly 80% had at least one episode of febrile neutropenia for which they were hospitalized^26^. These findings may mirror our study as we found at least 36% of NB-HR had one BSI despite the total BSI rate being 26.8%. Another study by Garaventa et al. found statistical differences in infectious risks, (5% versus 25% (P 0.011) between 2 two induction regimens for NB-HR Memorial Sloan Kettering Cancer Center N5 (MSKCC-N5) versus the rapid treatment (cisplatin [C], vincristine [O], carboplatin [J], etoposide [E], and cyclophosphamide [C], known as COJEC) COJEC respectively^26^. Regarding the rapid COJEC study, it is unclear what infectious etiologies were included in this study, which makes it challenging to compare it with our results.

VGS was determined to be the most isolated pathogen in this cohort of pediatric oncology patients with FN. This finding is similar to other studies in which VGS has been shown to account for the majority of first episodes of bacteremia in pediatric NB-HR patients throughout treatment^25^. The present study further demonstrated that patients with NB-HR were more likely to have VGS bacteremia during induction chemotherapy than patients with other solid tumors, thus highlighting that pediatric NB-HR patients are considered at higher risk for VGS bacteremia.

In a multivariate analysis, patients with mucositis were twice as likely to have VGS bacteremia, and both NB-HR during induction chemotherapy and mucositis were found to be predictors of VGS bacteremia. Treatment with high-dose cytosine arabinoside (HD-ARAC) is known to be associated with cytarabine-induced mucositis, prolonged neutropenia, and VGS bacteremia in AML pediatric patients^10,28–33^. However, a more recent study on AML showed that the high rate of VGS bacteremia was irrespective of the dose of cytarabine and rather dependent on the intensive chemotherapy treatment causing immunosuppression, cumulative mucosal damage, and sulfamethoxazole/trimethoprim prophylaxis^34^, the latter of which is believed to increase the frequency of VGS infections by allowing the overgrowth of resistant gram-positive organisms^16,30,35,36^. The induction chemotherapy regimen of NB-HR consists of five to six 5–6 cycles of intensive myelosuppressive chemotherapy including high-dose cyclophosphamide, etoposide, cisplatin, doxorubicin, and etoposide meant to alleviate the primary metastatic tumor burden^4^. Although this is not an AraC-based regimen, this regimen is associated with the high rate of VGS BSI in pediatric NB-HR during induction chemotherapy. These findings emphasize that NB-HR induction regimens involve intensive chemotherapy leading to both prolonged neutropenia between each cycle of induction and severe GI mucositis between each cycle of induction, which in turn leads to a higher VGS translocation rate to the bloodstream during periods of severe neutropenia. Mucositis rates in NB-HR patients are higher than in those with other cancer types, including AML and SCT patients. Although the present study investigated BSI in NB-HR during induction chemotherapy patients with FN, other studies have found that neuroblastoma patients who present with non-neutropenic fever may also be at risk of bloodstream infections, although with lower rates of VGS bacteremia^37^. One study in non-neutropenic febrile patients with NB found only one case of VGS bacteremia out of 39 positive blood cultures^36^, which may underscore the role of profound neutropenia as an additional risk factor in VGS bacteremia.

Patients with NB-HR in this study had significantly more PICU admissions compared to patients with other solid tumors. This is consistent with findings from a similar study comparing NB-HR to other cancers, where 70% of the patients had on average two additional admissions for infectious complications during induction when compared to other malignancies^26^. NB-HR during induction chemotherapy patients had higher FN admission rates between cycles compared with other solid tumors. This is consistent with previous studies where NB-HR has also been shown to be a risk factor for unplanned readmissions within 30 days of hospital discharge^38^. This increase in complications is likely linked to the increase in the to the increased intensity and number of chemotherapy cycles during the induction phase of NB-HR treatment. The prolonged central venous catheter uses in this population, particularly externalized catheters, could also expose patients to increased infection risk^26,37^. This high intensity of upfront therapy and central venous catheter use in NB-HR during induction chemotherapy highlights the importance of separating it from other solid tumors when considering the risk of infection.

The present study is limited by the retrospective nature of its design. As such, other potential risk factors for bacteremia in this patient population, including G-CSF administration^20,39^, antibiotic prophylaxis^36,40^, type of central venous catheter^26,37^, and the treatment of bacteremia episodes, which was at the discretion of the primary team, cannot be fully addressed. Additionally, this is a single-center study in an urban city, so these results may not be generalizable to other environments. Despite its limitations, this study includes a relatively large cohort (82 patients) of NB-HR patients during induction chemotherapy with FN and is the first to compare outcomes and infections in pediatric NB-HR patients during induction chemotherapy with those in patients with other cancer types. These This study’s findings led to an institutional change to empirical antibiotics of FN in NB-HR patients during induction to cefepime given a high rate of VGS given ceftazidime has poor VGS coverage. Also, given NB-HR during induction chemotherapy is more intensive with frequent neutropenia intervals during induction chemotherapy, so patients and their families should be educated about potential infectious burdens and complications during the induction phase to avoid any delay from SCT and improve overall outcomes. Future prospective studies are still needed to confirm these results and to further delineate risk factors for and understand the etiology of VGS bacteremia in this patient population.

## Conclusion

NB-HR during induction chemotherapy patients during induction chemotherapy typically receives high-intensity of treatment, which may render them to be more severely and chronically immunocompromised and thereby predisposed to infections, particularly VGS bacteremia. It is advisable to start distinguishing NB-HR from other solid tumors due to the worse outcomes Further research is needed to define the infectious risk of NB-HR during induction chemotherapy and so forth to improve their outcomes.

## Data Availability

The datasets used and/or analyzed during the current study available from the corresponding author on reasonable request.

## Abbreviations

FN: Febrile neutropenia
SCT: Stem cell transplantation
ST: Solid tumors
NB-HR: Neuroblastoma high risk
AML: Acute myelogenous leukemia
ALL: Acute lymphocytic leukemia
FNE: Febrile neutropenia episode
VGS: Viridans group streptococci
PICU: Pediatric intensive care unit
NB: Neuroblastoma
INRG: International neuroblastoma risk group
CLABSI: Central line associated bloodstream infections
BSI: Blood stream infection
HSCT: Hematopoietic stem cell transplantation
VSSS: Viridans-related septic shock syndrome
UCM: University of Chicago medicine
ICD-9: International classification for disease-9th edition
EMR: Electronic medical record
NHSN: National health safety network
ANC: Absolute neutrophil count
ALC: Absolute lymphocyte count
AMC: Absolute monocyte count
URI: Upper respiratory tract infection
GP: Gram positive
BMT: Bone marrow transplant
HD-ARAC: High-dose cytosine arabinoside
GM-CSF: Granulocyte–macrophage colony-stimulating factor
